# Change in the corneal material mechanical property for small incision lenticule extraction surgery

**DOI:** 10.3389/fbioe.2023.1034961

**Published:** 2023-02-20

**Authors:** Wenjing Gao, Xinheng Zhao, Yan Wang

**Affiliations:** ^1^ Clinical College of Ophthalmology, Tianjin Medical University, Tianjin, China; ^2^ Tianjin Key Lab of Ophthalmology and Visual Science, Tianjin Eye Institute, Tianjin Eye Hospital, Tianjin, China; ^3^ Nankai University Eye Institute, Nankai University Affiliated Eye Hospital, Nankai University, Tianjin, China

**Keywords:** stress-strain index, cornea, material property, biomechanical property, small incision lenticule extraction surgery, myopia

## Abstract

**Purpose:** To assess the distribution characteristics and related factors of stress-strain index (SSI) values and discuss changes in biomechanical parameters, including SSI, after small incision lenticule extraction (SMILE) surgery.

**Methods:** This study included 253 patients who underwent SMILE (253 eyes). SSI and other biomechanical parameters were measured using corneal visualization Scheimpflug technology before and 3 months after surgery. The data collected included SSI, central corneal thickness (CCT), and eight other dynamic corneal response parameters. The Kolmogorov–Smirnov test, Pearson and partial correlation analyses, and paired-sample *t*-tests were used for statistical analyses.

**Results:** Both pre-op SSI and ΔSSI follow a normal distribution, while post-op SSI does not follow a normal distribution. The decline in SSI after SMILE surgery was not statistically significant, and the data dispersion of SSI after SMILE surgery was close to that before surgery (*p* > 0.05). No statistical correlation was noted between SSI values and age and pre-op CCT (all *p* > 0.05). However, both pre- and post-op SSI values decreased with increasing degree of myopia (all *p* < 0.05), and weakly correlated with preoperative intraocular pressure and biomechanically corrected intraocular pressure (all *p* < 0.05). Other biomechanical parameters changed significantly after surgery (all *p* < 0.001). After SMILE, the magnitude of the deformation at the highest concave, deformation ratio, and integral radius increased significantly (all *p* < 0.001), while the Ambrosio relational thickness horizontal, stiffness parameter A1, and Corvis biomechanical index decreased significantly (*p* < 0.001).

**Conclusion:** SSI, which reflects essential corneal material attributes, differs from other corneal biomechanical parameters and remains stable before and after SMILE surgery, and can be used as an indicator to evaluate changes in corneal material properties after SMILE surgery.

## 1 Introduction

Ectasia is a severe postoperative complication of corneal refractive surgery and is considered the result of corneal biomechanical decompensation (Randleman et al., 2008). The minimally invasive cap-based refractive surgery small incision lenticule extraction (SMILE) has been proposed to preserve corneal integrity and mechanical properties better than flap-based surgery, because of the fiber-sparing incision of the strong anterior corneal lamellae (Raevdal et al., 2019). However, there are still a few reports of corneal ectasia after SMILE (Wu et al., 2014; Mattila and Holopainen, 2016; Raevdal et al., 2019; Brar et al., 2021; Shang et al., 2021). Therefore, it is of great significance to explore the changes in the biomechanical properties of the cornea after SMILE surgery. Previous studies (He et al., 2022; Xin et al., 2022.) have found significant differences in biomechanical parameters after SMILE surgery, such as the Ambrosio relational thickness horizontal (ARTh) and stiffness parameter A1 (SP-A1). However, these studies have not clarified whether the cause of the ectasia is related to changes in the properties of the corneal material itself, and the changes in the stress-strain index (SSI) that represent the properties of corneal materials after SMILE have not been reported.

The stress-strain behavior of biological tissues such as the cornea is non-linear (Elsheikh et al., 2007), so the tangent modulus, the measure of corneal material stiffness, is not a constant value but changes with changes in stress and strain. Previous studies have also shown that most corneal biomechanical metrics provided by the corneal visualization Scheimpflug technology (Corvis ST) are strongly related to central corneal thickness (CCT) and intraocular pressure (IOP). Recently, Eliasy et al. (2019) simulated the effect of IOP and Corvis ST air puff with a finite element numerical model. To evaluate the material mechanical behavior of the cornea, they established an algorithm for the tangent modulus, which is a measure of material stiffness under any IOP, and obtained an SSI, less dependent on IOP and corneal geometry. Previous studies have attempted to measure the corneal mechanical properties *in vivo*. These include SP-A1 and stiffness parameter highest concavity (Roberts et al., 2017), which are related to the diagnosis of keratoconus and significantly increase after corneal crosslinking; however, they cannot provide corneal material behavior measurements that are less correlated with geometry and IOP. In postoperative corneal tissue without pathological changes, SSI is primarily considered to be different from stiffness parameters in characterizing the real material mechanical properties of the cornea per unit thickness. Both simulated and clinical keratoconic corneas (Zhang et al., 2021) demonstrated substantial reductions in SSI values inside the cone. These SSI reductions depended on the extent of the disease and increased with more considerable simulated losses in fibril density within the cone area, whereas the SSI increased after corneal crosslinking surgery (Bao et al., 2021). The SSI values and their regional variations showed minimum change with alterations in corneal thickness, IOP, and curvature. It has been proven that SSI can be used to evaluate the attributes of corneal material.

As a viscoelastic biological tissue, it is necessary to consider the changes in corneal material attributes after SMILE. Influencing factors may include inflammation reaction (Kim et al., 2006), fiber healing responses, and cutting depth, among others (Sonigo et al., 2006; Shetty et al., 2017). This study aimed to evaluate the distribution characteristics, variations, and related factors of SSI values before and after SMILE surgery, to help manage eye disease and predict surgical outcomes, and to provide guidance for safe surgical cutting from the perspective of material properties.

## 2 Materials and methods

### 2.1 Study design

The records of patients who underwent SMILE were examined at the corneal refractive surgery center of Tianjin Eye Hospital. The inclusion criteria included an age of 18 years or older, stable refraction (change of ±0.50 diopters [D] or less) for at least 2 years before the operation, spherical refraction from −0.50 to −8.00 D, astigmatism less than −5.00 D, corrected distance visual acuity of 20/25 or better, and CCT greater than 480 µm. Patients were asked to discontinue soft contact lenses for 2 weeks and rigid gas-permeable contact lenses for 4 weeks before surgery. The exclusion criteria included ocular trauma, a history of ophthalmic surgery, glaucoma, keratoconus, diabetes, abnormal immune function, and systemic connective tissue disease.

The sample size calculation uses the formula:

$$n={\left[\left(\mu_{\alpha }+\mu_{\beta }\right)/\left(\frac{\delta }{\sigma }\right)\right]}^{2}+\frac{1}{2}*\mu_{\alpha }^{2}$$

n is the required sample size, *σ* is the total standard deviation, and *δ* = $\mu_{0}$ - $\mu_{1}$ , with $\mu_{0}$ being the known total mean, and $\mu_{1}$ the total mean of the experimental results. $\mu_{\alpha }$ and $\mu_{\beta }$ are the boundary values of the standard normal distribution corresponding to the significance level *α* and type II error probability *β*, respectively.

Where *δ* = 0.03, *σ* = 0.13, bilateral *α* = 0.05, $\mu_{0.05/2}$ = 1.96, *β* = 0.1, and $\mu_{0.01}$ = 1.282, the final calculation is n = 200.

The study collected data on 253 patients, and all of them underwent a complete ophthalmological examination between 09:00–11:00 a.m. All participants provided informed consent for the use of their data for research. The studies involving human participants were reviewed and approved by the Medical Ethics Committee of Tianjin Eye Hospital. The participants provided their written informed consent to participate in this study. This study adhered to the tenets of the Declaration of Helsinki.

### 2.2 Surgical procedure

All procedures were performed by the same experienced surgeon (YW). SMILE was performed using the VisuMax femtosecond laser system (Carl Zeiss Meditec AG, Jena, Germany) through a 2-mm tunnel incision at the 12-o’clock position with 130 nJ of energy. The lenticule was created as follows: Posterior surface (from the periphery to the center), border, anterior surface (from the center to the periphery), and side-cut incision. The cap diameter was 7.3–7.6 mm, and the cap thickness was 110–120 µm. The lenticule diameter was 6.3–6.6 mm. A blunt spatula is often used to separate the stromal lenticule before its removal through the tunnel incision. Levofloxacin 0.5% eye drops (Carivid; Santen) were instilled four times a day for 3 days before surgery. Postoperatively, topical levofloxacin 0.5% (Carivid; Santen) was prescribed four times a day for 3 days. Fluoroethylene 0.1% eye drops (Flumetholon; Santen) were prescribed four times daily and tapered off every 2–3 weeks. Artificial tear drops were administered four times a day for 3 months.

### 2.3 Biomechanical evaluation

Corneal biomechanical parameters were obtained using Corvis ST analyzer (Oculus, Germany). Corvis ST is a visual dynamic IOP analyzer that integrates ultra-high-speed Seheimpflug technology into a non-contact IOP measuring instrument to study the entire dynamic process of corneal deformation under external forces. The dynamic corneal response parameters were analyzed and recorded. *Δ* indicates the difference between the pre- and post-SMILE values. All Corvis ST examinations were acquired with good quality scores. Only the right eye was included in the analysis to avoid any bias in the relationship between the bilateral eyes (Ying et al., 2018). The same experienced clinician (XZ) took all measurements using the same instruments. The instrument was calibrated by a technician before initiating the study.

Several dynamic corneal response parameters were evaluated including CCT, IOP, biomechanically corrected intraocular pressure (bIOP), deformation amplitude at the highest concavity (HC Deformation Amp), deformation (DA) ratio, Ambrosio relational thickness horizontal (ARTh), integrated radius (IR), SP-A1, and Corvis biomechanical index (CBI). The SSI used to evaluate the corneal material stiffness was also included.

### 2.4 Statistical analysis

Statistical analyses were performed using the SPSS statistical package 26 (SPSS, IBM, Chicago, IL, United States). Descriptive statistical results included means, standard deviations, and the minimum and maximum values of the parameters. The Kolmogorov–Smirnov test was used to assess the normal distribution of the data. Paired-sample t-tests were used to analyze the differences between the pre-and post-SMILE biometric values. Correlations between SSI and different parameters were analyzed using Pearson’s or Spearman’s correlation. Statistical significance was set at *p* < 0.05.

## 3 Results

### 3.1 Basic information for participants

A total of 253 right eyes from 253 participants were included in the study. The mean age of all participants was 24.0 ± 5.9 years (range: 18–45 years). The study participants were 135 men and 118 women. The pre- and post-operative ocular biological parameters of the participants, including spherical diopter, cylindrical diopter, spherical equivalent refractive (SEQ), and uncorrected distance visual acuity (UDVA), corrected distance visual acuity (CDVA), IOP, and bIOP, are shown in Table 1. The Corvis ST results before and after SMILE surgery, along with the differences in main metrics and the paired-sample *t*-test results, are presented in Table 2.

**TABLE 1:** Ocular biological parameters before and after small incision lenticule extraction surgery.

Parameters	Pre		Post	
	Mean ± SD	Range	Mean ± SD	Range
Sphere (D)	−5.069 ± 1.664	−9.750–−0.500	−0.100 ± 0.211	−1.250–0.500
Cylinder (D)	−0.796 ± 0.676	−3.250–0.000	−0.089 ± 0.177	−0.750–0.250
SEQ (D)	−5.466 ± 1.734	−10.125–−1.250	−0.144 ± 0.218	−1.250–0.250
UDVA (logMAR)	0.661 ± 0.281	0.200–1.000	−0.113 ± 0.085	−0.300–0.150
CDVA (logMAR)	0.001 ± 0.014	−0.100–0.100	−0.137 ± 0.068	−0.300–0.000
IOP, mmHg	16.607 ± 2.176	12.000–25.000	13.043 ± 2.359	7.000–32.500
bIOP, mmHg	16.180 ± 1.871	12.000–21.700	15.122 ± 2.066	9.600–29.300

D = diopters; SEQ, spherical equivalent refraction; UDVA, uncorrected distance visual acuity; CDVA, corrected distance visual acuity; bIOP, biomechanically corrected IOP.

**TABLE 2:** Corneal visualization Scheimpflug technology (Corvis ST) metrics were collected before and after small incision lenticule extraction surgery.

Metrics	Pre		Post		Difference			
	Mean ± SD	Range	Mean ± SD	Range	Mean ± SD	Range	t	*p*
SSI	0.842 ± 0.128	0.438–1.299	0.838 ± 0.135	0.450–1.475	−0.004 ± 0.117	−0.363–0.372	−0.508	0.612
CCT, μm	556.217 ± 30.093	490.000–657.000	451.395 ± 41.936	348.000–590.000	−104.822 ± 29.471	−189–35	−56.574	<0.001
HC Deformation Amp, mm	1.089 ± 0.091	0.869–1.356	1.153 ± 0.097	0.800–1.462	0.065 ± 0.083	−0.227–0.408	179.104	<0.001
DA ratio	4.409 ± 0.885	3.230–16.363	5.673 ± 0.754	3.002–11.807	1.263 ± 1.047	−10.921–7.893	19.185	<0.001
ARTh	562.532 ± 100.713	331.264–918.044	188.565 ± 67.132	71.354–441.706	−373.967 ± 103.921	−776.427–6.615	−57.239	<0.001
IR, mm-1	7.966 ± 0.910	4.997–10.636	10.381 ± 1.246	4.403–13.502	2.415 ± 0.981	−2.541–4.782	39.158	<0.001
SP-A1, mmHg/mm	109.895 ± 15.065	68.368–166.672	81.981 ± 17.291	36.498–168.729	−27.914 ± 14.810	−104.882–35.307	−29.98	<0.001
CBI	0.029 ± 0.101	0.000–1.000	0.969 ± 0.126	0.000–1.000	0.940 ± 0.157	0.000–1.000	95.44	<0.001

SD, standard deviation; SSI, stress-strain index; CCT, central corneal thickness; HC, deformation Amp, deformation amplitude at the highest concavity; DA, ratio, deformation ratio; ARTh, Ambrosio relational thickness horizontal; IR, integrated radius; SP-A1, stiffness parameter at first applanation; CBI, corvis biomechanical index.

### 3.2 Distribution characteristics and change in SSI after SMILE surgery

**FIGURE 1:**
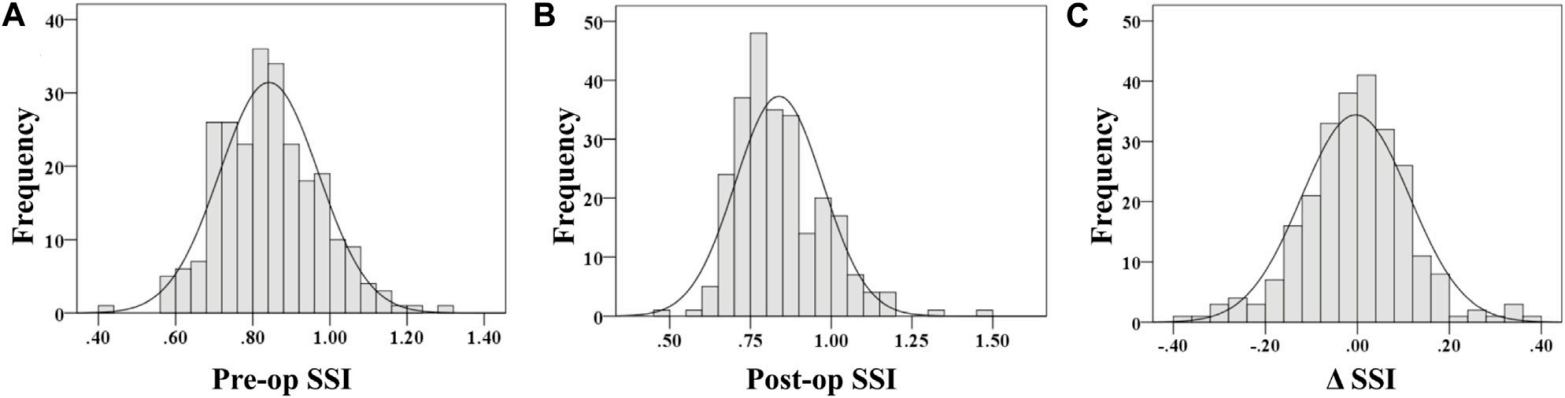
Distribution of pre-op stress-strain index **(A)**, post-op stress-strain index **(B)** and Δ stress-strain index **(C)**.

**FIGURE 2:**
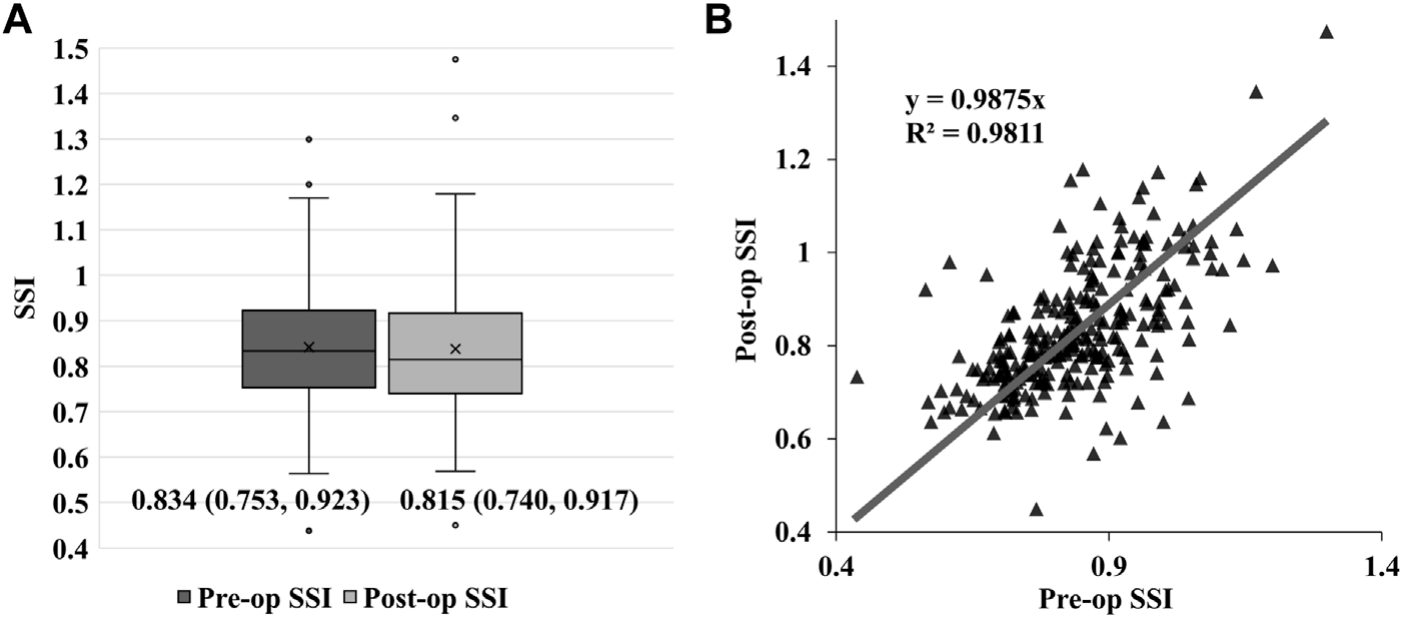
Box-plot graph of the pre-and post-op stress-strain index (SSI) values. The bar inside each box represents the median, and each box extends from the 25th percentile to the 75th percentile distribution in each group. The median of the post-op SSI values is lower than the pre-op SSI values without statistical difference (*p* > 0.05), and the two groups have a comparable data spread **(A)**. Scatter diagram and linear fit for pre- and post-op SSI values, post-op SSI = 0.988×Pre-op SSI, *R* ^2^ = 0.981 **(B)**.

The SSI values of the 253 participants before surgery were normally distributed (Kolmogorov–Smirnov test, *p* = 0.2 Figure 1A), with an average of 0.842 ± 0.128. After SMILE the SSI values dropped to 0.838 ± 0.135. The right-sided distribution showed a non-normal distribution (Kolmogorov–Smirnov test, *p* < 0.001 Figure 1B). However, the decline in SSI after SMILE surgery was not statistically significant, and was normally distributed (p > 0.05, Figure 1C) and the data spread of SSI after the operation was close to that before surgery (Figure 2A). The pre- and post-op SSI was highly correlated (post-op SSI values = 0.988 * Pre-op SSI values, *R* ^2^ = 0.981) (Figure 2B).

### 3.3 SSI values with their relevant factors

There was no statistical correlation between pre- and post-op SSI values with age and pre-op CCT (*p* > 0.05). However, pre- and post-op SSI values were associated with ΔCCT (*p* < 0.05), and weakly correlated with IOP and bIOP before surgery (*p* < 0.05). Other biomechanical parameters, including HC Deformation Amp., DA ratio, ARTh, IR, and SP-A1 were all associated with pre-op CCT (*p* < 0.05), and HC Deformation Amp., DA ratio, IR, and SP-A1 were all moderately or strongly correlated with preoperative IOP and bIOP (*p* < 0.001) (Table 3).

**TABLE 3:** Pearson’s or Spearman’s correlation analysis of the factors related to stress-strain index values and other biomechanical parameters.

		Age, year	Pre-sphere, D	Pre-cylinder, D	Pre-SEQ, D	Pre-op CCT, μm	ΔCCT, μm	Pre-op IOP, mmHg	Pre-op bIOP, mmHg
Pre-op SSI	R	0.098	.323**	.229**	.354**	0.045	−.365**	.257**	.256**
	*p*	0.12	<0.001	0.001	<0.001	0.475	<0.001	<0.001	<0.001
Post-op SSI	R	0.084	.218**	.233**	.254**	−0.091	−.39**	.258**	.258**
	*p*	0.182	0.002	0.001	<0.001	0.148	<0.001	<0.001	<0.001
HC Deformation Amp., mm	R	.253**	−0.031	−0.039	−0.037	−.257**	0.106	−.804**	−.764**
	*p*	<0.001	0.656	0.58	0.593	<0.001	0.091	<0.001	<0.001
DA ratio	R	0.013	−0.071	−0.022	−0.073	−.227**	0.081	−.302**	−.229**
	*p*	0.836	0.305	0.751	0.296	<0.001	0.197	<0.001	<0.001
ARTh	R	−0.051	−0.04	−0.052	−0.049	.461**	0.051	0.107	−0.075
	*p*	0.416	0.566	0.452	0.486	<0.001	0.42	0.089	0.236
IR, mm^-1^	R	0.091	−0.065	−.163*	−0.094	−.465**	.172**	−.552**	−.395**
	*p*	0.148	0.354	0.018	0.178	<0.001	0.006	<0.001	<0.001
SP-A1, mmHg/mm	R	−.205**	−0.053	−0.065	−0.064	.696**	0.02	.738**	.496**
	*p*	0.001	0.444	0.352	0.359	<0.001	0.75	<0.001	<0.001
CBI	R	−0.027	−0.048	−0.053	−0.056	−.252**	0.074	−.159*	−0.065
	*p*	0.671	0.495	0.447	0.422	<0.001	0.243	0.012	0.3

Notes: ** represents *p* < 0.01, * represents *p* < 0.05. D, diopters; SEQ, spherical equivalent refraction SSI, stress-strain index; CCT, central corneal thickness; HC, deformation Amp, deformation amplitude at the highest concavity; DA, ratio, deformation ratio; ARTh, Ambrosio relational thickness horizontal; bIOP, biomechanically corrected IOP; IR, integrated radius; SP-A1, stiffness parameter at first applanation; CBI, corvis biomechanical index.

### 3.4 Other biomechanical parameters after surgery

Other biomechanical parameters showed significant changes after surgery (*p* < 0.001). IOP, bIOP, ARTh, SP-A1, and CBI were significantly reduced, whereas HC Deformation Amp., DA ratio, and IR were significantly increased after SMILE (*p* < 0.001) (Table 1).

**FIGURE 3:**
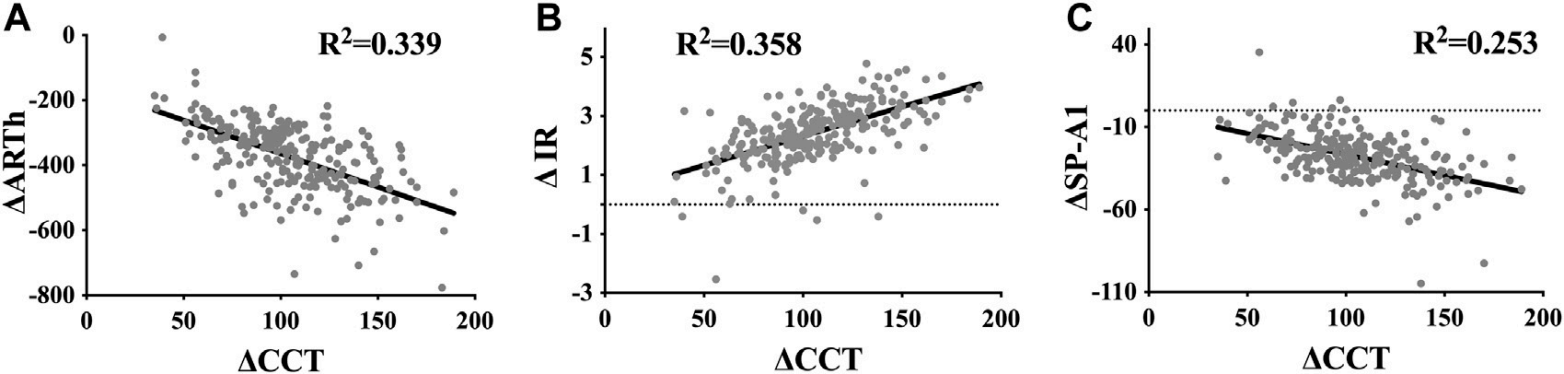
Scatter diagram and linear fit for the differences in dynamic corneal response parameters, including ARTh **(A)**, IR **(B)**, and SP-A1 **(C)**, recorded before and after small incision lenticule extraction, and the corresponding changes in central corneal thickness.

ΔCCT was moderately correlated with ΔARTh (*R* ^2^ = 0.339) and ΔIR (*R* ^2^ = 0.358), and weakly correlated with ΔSP-A1 (*R* ^2^ = 0.253) (Figures 3A–C). However, there were no correlations between ΔCCT, ΔHC Deformation Amp. (*R* ^2^ = 0.059), and *Δ* DA ratio (*R* ^2^ = 0.043).

## 4 Discussion

To date, the criterion for corneal biomechanical screening strategy, which is warranted to prevent corneal ectasia after corneal refractive surgery, has not been determined. It is therefore necessary to further explore the factors that influence corneal biomechanical properties after SMILE. To the best of our knowledge, this is the first study to compare the corneal material properties before and after SMILE using SSI. Our study complemented the changes in corneal material mechanical properties after SMILE surgery, provided sufficient corneal material property data for finite element numerical models, and provided a new basis for exploring the mechanism of corneal ectasia after refractive surgery.

The results in our study were consistent with those of previous studies (Eliasy et al., 2019; Liu et al., 2020). SSI was not related to CCT, and only weakly correlated with IOP, which is significantly different from other biomechanical parameters reported by Corvis ST, HC Deformation Amp., DA ratio, ARTh, IR, and SP-A1. CBI cannot exclude the influence of CCT during the calculation process, it is greatly affected by IOP, and does not reflect the characteristics of the material itself, while SSI is relatively stable. Therefore, it is more promising to explore the effects of differences in the internal structure of corneal materials on biomechanics through SSI. For example, the localized aspect of the microstructure deterioration in keratoconus boosted the need to map the corneal stiffness to identify the regional biomechanical failure through SSI (Lopes and Elsheikh, 2022; Lopes et al., 2022). In that regard, it has been observed through SSI maps that the regional deterioration in stiffness was concerted inside the pathological keratoconus region.

In contrast, only mild non-significant alterations were observed outside the area of pathology (Lopes et al., 2022). Martínez-Sánchez et al. (2022) found that SSI decreases significantly after topical prostaglandin (PG) therapy. Additionally, topical PG therapy reduced corneal stiffness, suggesting that the ocular hypotensive effect of these drugs is overestimated if we measure IOP with Goldman Applanation Tonometry (GAT) instead of bIOP. Because SSI can truly reflect the differences in material properties, one study (Vinciguerra et al., 2022) evaluated the difference between Caucasian and healthy Chinese subjects in Corvis ST dynamic corneal response parameters and found a significant difference in SSI values.

However, our findings that SSI is not age-related, are inconsistent with the results of the study by Eliasy et al. (2019). This may be because our study included young people and the age range was limited, with an average age of 24.0 ± 5.9 years (range: 18–45 years). Liu et al. (2020) also found no significant correlation between SSI and age, for individuals aged younger than 35 years.

This study observed a slight decrease in SSI values from 0.842 to 0.838 after SMILE surgery, which was not associated with statistical difference (*p* > 0.05). Other biomechanical parameters, including HC Deformation Amp., DA ratio, ARTh, IR, SP-A1, and CBI, all underwent significant changes after SMILE (*p* < 0.001). This is consistent with the study by (Kenia et al., 2021) that compared the SSI 1 month after laser *in situ* keratomileuses (LASIK) and femtosecond LASIK and found that its reduction was not statistically significant in either group.

Although the decline in SSI was not statistically significant and the data dispersion of SSI after SMILE surgery was close to that before surgery, both pre-op SSI and ΔSSI were in line with normal distribution (Figures 1A, C). At the same time, post-op SSI was associated was non-normally distributed (Figure 1B), which suggests that the effect of surgery on corneal SSI cannot be ignored entirely.

It should be emphasized that the lack of significant differences pre- and post-op does not imply that the material properties of the cornea are the same. In this study, scatter diagrams and linear fit for pre- and post-op SSI values indicated that the effect of different healing responses in individuals on SSI could not be ignored. Multiple studies have shown that such changes exist. Shetty et al. (2017) have shown that collagen fiber remodeling occurs at the incision surface for SMILE, with increased micro-deformations of the pure elastic layer and increased collagen fiber crimping. The strong interaction between the laser and the material may lead to stress redistribution on the cutting surface, thus affecting material stiffness (Wollensak et al., 2014). Wang et al. (2020) found that when a femtosecond laser is applied to collagen tissue, low-density plasma produces reactive oxygen species, which form a crosslinking reaction with surrounding proteins, and the spatial distribution of mechanical properties changes. Noticeable inflammatory reactions may occur during the early stages after femtosecond laser cutting (Kim et al., 2006), and more fibrotic scars can be seen in the peripheral area under confocal microscopy (Sonigo et al., 2006). However, collagen fiber remodeling after SMILE may only occur near the cut surface, and most other corneal tissues are unaffected. Overall, the impact of collagen fiber remodeling on the corneal material properties of the cutting surface requires further study.

This study found that SSI before and after SMILE surgery was significantly correlated with spherical diopter, cylindrical diopter, and equivalent spherical diopter in myopic patients. The higher the degree of myopia or astigmatism, the lower the SSI value and the lower the stiffness of the corneal material, which is consistent with results reported by Han et al. (2020) for myopia. This suggests that we should pay more attention to the mechanical properties of corneal materials in patients with high myopia or high astigmatism, strictly screen for preoperative SSI, and closely follow up the degree of SSI changes after surgery to prevent complications such as corneal ectasia from affecting postoperative visual quality.

Our results confirmed that preoperative and postoperative SSI values are both negatively correlated with the cutting thickness. The cutting thickness reflects the diopter. As the degree of myopia increases the cutting thickness increases, thereby further confirming that with the increase of myopia the mechanical properties of corneal materials become worse.

We also found that preoperative SSI might be a significant predictor of postoperative SSI. Postoperative SSI was correlated with cutting thickness, suggesting that patients with high diopters tend to have lower preoperative SSI, and postoperative SSI values are still low. In addition, the risk of corneal ectasia after surgery may be higher due to the large cutting thickness. Thus, more attention should be paid to SSI in patients with high myopia during preoperative screening, to evaluate the risk of surgery and comprehensively prevent corneal ectasia.

Except for SSI, our study found that other biomechanical parameters, such as ΔARTh and ΔIR, were strongly correlated with cutting thickness. This is consistent with the results of Liu and others (Liu et al., 2021), who found that ARTh after femtosecond LASIK and laser-assisted sub-epithelial keratectomy was significantly correlated with cutting thickness. IR represents the radius of global deformation, which can represent the overall anti-deformation ability of the cornea. Our study showed that ΔIR was positively correlated with ΔCCT. Greater cutting thickness was associated with higher IR and worse overall anti-deformation ability of the cornea. This suggests that we should pay attention to the cutting thickness, to prevent excessive reduction of the overall anti-deformation ability of the cornea and corneal ectasia as much as possible.

Diurnal changes may influence the corneal thickness and IOP (Read et al., 2008; Read and Collins, 2009). The corneal thickness may increase when one just wakes up in the morning due to edema, and the IOP may decrease in the afternoon. All these changes may potentially affect results on SSI. Thus, in order to avoid the impact of such diurnal changes on the results of this study, all patients were examined between 09:00–11:00 a.m.

The main limitation of the present study was its short follow-up duration. Some studies have proven that the biomechanical properties of the cornea are stable 3 months after SMILE (Mastropasqua et al., 2014; Wu et al., 2014); however, changes in SSI still need to be observed over the long term. We also did not include follow-up data at 1 month after surgery, considering that there may be a healing response in the first month after surgery that could result in instability of SSI. In addition, this study mainly included younger patients (mean age: 24.0 ± 5.9 years) because most of the people who undergo SMILE surgery in our country are young patients because of the requirements to join the military or some jobs. People over 45 years old rarely receive SMILE surgery, because of possible accommodation problems such as presbyopia. It is well-known that there is a direct relationship between SSI and age. Therefore, further studies involving patients with a broader age range undergoing SMILE surgery should be conducted.

In conclusion, SSI is more stable than other biomechanical parameters for assessing corneal material properties after SMILE. Exploring changes in biomechanical parameters, including SSI after surgery, aids in comprehensively evaluating the risk of corneal ectasia after SMILE. In the future, a stress-strain map may help us detect ectasia after corneal refractive surgery that begins with localized corneal tissue.

## Data Availability

The original contributions presented in the study are included in the article/Supplementary Material, further inquiries can be directed to the corresponding author.

## Abbreviations

ARTh, Ambrosio relational thickness horizontal; bIOP, biomechanically corrected intraocular pressure; CBI, Corvis biomechanical index; CCT, central corneal thickness; DA, deformation; HC Deformation Amp, deformation amplitude at the highest concavity; IOP, intraocular pressure; IR, integral radius; SMILE, small incision lenticule extraction; SP-A1, stiffness parameter at first applanation; SSI, stress-strain index.
